# Failure to Replicate Depletion of Self-Control

**DOI:** 10.1371/journal.pone.0109950

**Published:** 2014-10-21

**Authors:** Xiaomeng Xu, Kathryn E. Demos, Tricia M. Leahey, Chantelle N. Hart, Jennifer Trautvetter, Pamela Coward, Kathryn R. Middleton, Rena R. Wing

**Affiliations:** 1 Department of Psychology, Idaho State University, Pocatello, Idaho, United States of America; 2 The Weight Control and Diabetes Research Center, The Miriam Hospital, Providence, Rhode Island, United States of America; 3 Department of Psychiatry and Human Behavior, Warren Alpert Medical School, Brown University, Providence, Rhode Island, United States of America; 4 Department of Public Health, Center for Obesity Research and Education, Temple University, Philadelphia, Pennsylvania, United States of America; Beijing Normal University, China

## Abstract

The limited resource or strength model of self-control posits that the use of self-regulatory resources leads to depletion and poorer performance on subsequent self-control tasks. We conducted four studies (two with community samples, two with young adult samples) utilizing a frequently used depletion procedure (crossing out letters protocol) and the two most frequently used dependent measures of self-control (handgrip perseverance and modified Stroop). In each study, participants completed a baseline self-control measure, a depletion or control task (randomized), and then the same measure of self-control a second time. There was no evidence for significant depletion effects in any of these four studies. The null results obtained in four attempts to replicate using strong methodological approaches may indicate that depletion has more limited effects than implied by prior publications. We encourage further efforts to replicate depletion (particularly among community samples) with full disclosure of positive and negative results.

## Introduction

Self-control, the effortful regulation of the self to overcome impulses and delay gratification, is an important capacity linked to success and better outcomes in a variety of domains including interpersonal relations, academics, and health [1]. One model of self-control [2]–[3] posits that self-control is a finite resource akin to muscle strength and is vulnerable to exhaustion after exertion. Depletion (sometimes referred to as ego depletion) occurs when a person's self-control resources have been exhausted, and thus performance on subsequent attempts at self-control is impaired. This model has been tested in a number of studies and a meta-analysis found support for a significant effect of ego depletion on hindering subsequent self-control task performance [4]. However, the majority of studies utilizing the strength model of self-control have been conducted with college-age students, which is problematic as the depletion effect may be specific to young adults, and may not generalize to other age groups [5]. Some have also questioned the existence of the depletion effect, suggesting that it is likely to be significantly overestimated due to publication bias [6].

Recently, attention has focused on developing interventions to strengthen self-control and buffer against depletion [7]. As part of such efforts, a reliable paradigm is needed to indicate whether interventions succeed in increasing self-control and decreasing the negative effects of depletion. The goal of the current investigation was to identify such a paradigm. We conducted two depletion studies with community samples and then repeated the two studies with young adult samples. We report here the results of these four studies with the goal of encouraging further efforts to replicate these paradigms with full disclosure of positive and negative results.

## Method

### Overview of Procedures

All four studies used a repeated measure design in which self-control was first assessed, followed by random assignment to a depletion task or control task, and then the assessment of self-control was repeated. The independent and dependent measures used in these studies were selected based on a meta-analysis of ego-depletion studies [4] and were chosen because they were the most frequently used tasks with consistently large effect sizes. The crossing out letters protocol (see detailed procedures below), was selected for the manipulation (Depletion vs. Control) task, as it was the most commonly used manipulation with a consistently large effect size (*d* = 0.77 across 20 studies). Handgrip persistence (*d* = 0.64 across 18 studies) and a modified Stroop task (*d* = 0.76 across 15 studies) were selected as the dependent measures for use in separate studies. Both dependent measures were tested first with community adults. The same protocols were then repeated with young adults, since the majority of prior studies were done with this age group [4] and there is some evidence that the depletion effect may be specific to young adults [5]. With a repeated measures design, an alpha of.05, and a 2-sided test of significance, samples of N = 40 (20 per group) for each study provided 91% power to detect large effects (d = 0.8) and 86% power to detect medium-large effects (d = 0.7) based on Cohen's standards [8]. These effects are consistent with effect sizes observed in prior studies with the crossing out letters protocol [4].

### Participants

Community adults and young adults were recruited using a large display board in a local hospital cafeteria or university dining hall, respectively, and by word of mouth. To be eligible for the study, community samples were required to be over the age of 18, with no upper age limit, and young adults to be aged 18–25. The only other eligibility criterion was to have fasted for two hours. This criterion was used to help control for glucose levels, as glucose may interfere with the depletion effect [9]. The research was approved by the Institutional Review Board (IRB) of The Miriam Hospital and in line with guidelines set by the IRB and The Declaration of Helsinki, all participants provided informed written consent. Participants received a $25 honorarium at the end of the study.

Fifty two community adults (18 men and 34 women), with a mean (± SD) age of 41.6±15.3 years were recruited for the handgrip protocol and 38 adults (11 men and 27 women) with a mean (± SD) age of 43.6±12.9 years for the modified Stroop protocol. There were no significant differences in age or ethnicity between the community participants who were randomly assigned to the depletion vs control condition in either protocol, all *p*s>.05.

Fifty young adults (32 men and 18 women), with a mean (±SD) age of 19.7±1.3 years were recruited for the handgrip protocol and 46 young adults (14 men and 32 women) with a mean (± SD) age of 21.2±2.8 years for the modified Stroop protocol. Those who were randomly assigned to the depletion condition did not significantly differ from those in the control condition on age or ethnicity, all *p*s>.05.

### Manipulation task (Depletion versus Control)

The depletion task as described by Baumeister et al. [2] has been used in many other studies [4]. Following the procedures reported by Baumeister et al [2], all participants were first given an easy task to complete, namely to cross out all instances of the letter “e” on printed pages of text for a period of two minutes. This task was used to establish a behavioral pattern. Subsequently, those who were randomized to the control group were instructed to continue to cross out every instance of the letter “e” on additional pages; those randomized to the depletion condition were given pages of text where the print was very light and were instructed to cross out all “e's” that were not adjacent to or one letter away from another vowel; this task required the use of self-control to override a previously learned behavioral pattern. Both depletion and control groups spent a total of 8 minutes on their task.

### Handgrip Persistence

Handgrip persistence was determined using a protocol described by Magen and Gross [10] and recommended by an expert in the field (R. Baumeister, personal communication, 2013). This protocol uses a hand dynamometer as the measuring device and examines persistence at 70% of the individuals' own maximum grip strength. This approach was selected over earlier protocols [11], that utilized a spring-based hand grip device (which could not be calibrated to the individual) and had participants squeeze it for as long as they could (measured based on how long it took for an object that was inserted between the springs to fall), without taking into account participants' maximum strength level.

As per Magen and Gross [10], the participants' maximum grip strength was first determined. Participants were seated with both feet on the floor, and instructed to hold the hand dynamometer (Lafayette Instruments Model 78010 Lafayette, Indiana) in their dominant hand with their elbow bent at a 45 degree angle such that the device was in their line of sight, and to squeeze the dynamometer as hard as they could for 3 seconds; this was used as participants' maximum grip. After a short break, participants were instructed to grip the dynamometer as they had before, squeeze it at or above 70% of their maximum grip strength and hold it for as long as possible. The instructions were read to each participant to minimize variability and the 70% point was clearly indicated on the dial so that participants were able to see the level at which they needed to maintain their grip. Hand grip persistence was assessed as time in seconds using a stop watch.

After completing the depletion/control task, all participants were again asked to hold the hand dynamometer at 70% of their maximum grip strength for as long as possible and persistence was again assessed.

### Modified Stroop

Versions of the modified Stroop computer task have been used extensively in previous literature, and the version used herein was based on the protocol reported in prior depletion studies [9], [12]–[13]. The task was conducted using Eprime Stimulus Presentation Software running on a laptop computer. Participants viewed color words (i.e., ‘red’, ‘yellow’, ‘green’, ‘blue’) that appeared one at a time in an incongruent font color (e.g., ‘red’ may be displayed in blue font) and the participant responded by pressing the key corresponding to the font color rather than the word itself. Each participant was given a brief practice round to orient them to the modified Stroop task, after which they completed 20 self-paced trials.

Upon completion of either the control or depletion protocol, all participants completed a second set of the modified Stroop task consisting of 80 trials, as has been done in prior studies [9], [13]. The primary outcome measure was the change in reaction time on the correct trials of the Stroop task from pre-to-post; the secondary outcome was pre-to-post change in the number of correct trials.

### Statistical Analyses

For both community and young adult samples, repeated measure analyses of variance were conducted, comparing the effects of the depletion and control conditions on self-control performance from pre-test to post-test. The primary outcome measure was the Condition X Time interaction. Results are presented as partial eta squares with the magnitudes of effect sizes corresponding to Small  = .01, Medium  = .06, and Large  = .14 [8]. To improve interpretation and comparison to previous studies, we further calculated mean differences, Cohen's d, and confidence intervals around the mean differences between the depletion and control groups, using pre-to-post change scores.

## Results

As described above, the two protocols were first tested in community samples and then repeated using college-aged young adult samples. However, for ease of presentation, we present the results for the handgrip perseverance protocol in the two different samples and then present the findings for the Stroop protocol.

### Handgrip Persistence (see Figure 1)

**Figure 1:**
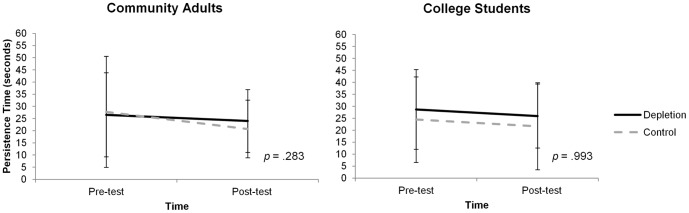
Mean (± SE) number of seconds that the dynamometer was held at pre-test and post-test in community adults and young adults by condition. (Depletion  =  solid line, Control  =  dashed line).

Within the community adults, hand grip persistence decreased from pre- to post-testing (Mean ± SE change of −4.96±2.08 s, *F*(1,50) = 5.28, *p* = .026, 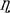 ^2^_partial_ = . 096), but there was no evidence that these changes differed between the DEPLETION and CONTROL conditions [mean ± SE change of −2.52±2.63 and −7.04±3.12 seconds, respectively, *F*(1,50) = 1.178, *p* = .283, 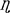 ^2^_partial_ = .023, mean difference  = −4.52±4.16, *d* = −.302, 95% CI of mean difference: −12.88, 3.84].

Likewise, there was a trend toward pre-to-post decreases in hand-grip strength in the young adults (−2.78±1.38 s, *F*(1, 47) = 3.96, *p* = .052, 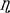 ^2^_partial_ = .078), but changes in hand grip persistence in this sample did not differ between the DEPLETION and CONTROL conditions [mean ± SE change in DEPLETION  = −2.77±2.10 s, and in CONTROL  = −2.79±1.77 s, *F*(1,47) = 0.001, *p* = .993, 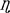 ^2^_partial_<.001, mean difference  = −0.02±2.79, *d* = −.002, 95% CI of mean difference: −5.64, 5.59]. Combining the data from both community adults and young adults again indicated no difference between the depletion and control group [mean ± SE change in persistence DEPLETION  = −2.65±1.65 s, CONTROL  = −5.12±1.90 s, *F*(1,99) = .963, *p* = .329, 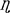 ^2^_partial_ = .010, mean difference  = −2.47±2.52, *d* = −.195, 95% CI of mean difference: −7.48, 2.53].

### Modified Stroop (see Figure 2)

**Figure 2:**
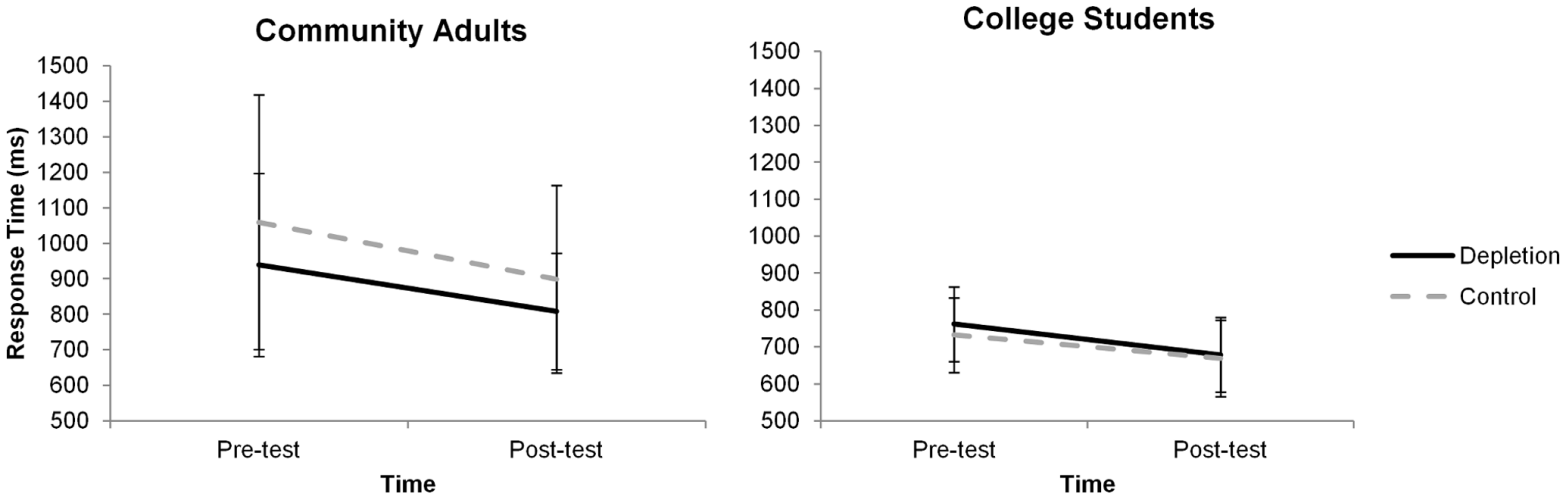
Mean (± SE) response time (in milliseconds) for Stroop responses at pre-test and post-test in community adults and young adults, by condition. (Depletion  =  solid line, Control  =  dashed line).

Within the community adults, reaction time on the Stroop task improved (i.e. decreased) from pre- to post-treatment [mean ± SE change  = −147.38±33.50, *F*(1,36) = 18.31, *p*<.001, 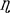 ^2^_partial_ = .337]. However, there were no significant differences in changes in reaction time (RT) for the DEPLETION versus the CONTROL group [mean ± SE change in RT DEPLETION  = −130.12±28.21 ms, mean ± SE change in RT CONTROL  = −161.34±56.75 ms, *F*(1,36) = .210, *p* = .649, 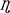 ^2^_partial_ = .006; mean difference  = −31.22±68.11 ms, *d* = −.149, 95% CI of mean difference: −169.35, 106.91].

Likewise, for young adults, reaction time on the Stroop task improved (i.e. decreased) from pre- to post-treatment [mean ± SE change  = −73.35±13.57 *F*(1,44) = 28.51, *p*<.001, 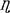 ^2^_partial_ = .393]. However, there were no significant differences in changes in RT for the DEPLETION versus the CONTROL group [mean change ± SE in RT DEPLETION  = −82.56±20.22 ms, mean change in RT CONTROL  = −63.30±18.08 ms; *F*(1,44) = .497, *p* = .485, 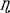 ^2^_partial_ = .011; mean difference  = 19.25±27.32 ms, *d* = .208, 95% CI of mean difference: −35.80, 74.31]. Further, there were no difference in changes in RT between the DEPLETION and CONTROL groups when the data from the community adults and young adults were combined, [mean change in RT DEPLETION  = −102.28±107.79 ms, mean change in RT CONTROL  = −111.18±107.79 ms, *F*(1,82) = .066, *p* = .798, 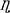 ^2^_partial_ = .001; mean difference  = −8.90±34.69 ms, *d* = −.056, 95% CI of mean difference: −77.92, 60.11]. Since 80 Stroop trials were conducted following the manipulation task, analyses were also done examining changes in each block of 20 trials. We observed no significant differences between the DEPLETION and CONTROL condition during any of the blocks of trials, all *ps*>.05. Similarly, no differences in changes in accuracy were observed between the DEPLETION and CONTROL group in either community adults or young adults, *p* = .330, 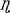 ^2^_partial_ = .026, and *p* = .915, 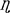 ^2^_partial_<.001, respectively.

**Figure 3:**
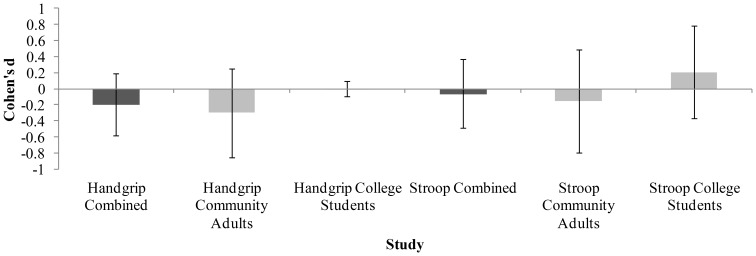
Mean effect sizes (Cohen's d) for each of the studies and combined samples, along with 95% confidence intervals.

For ease of comparison, we have presented the Cohens d (plus 95% CI around d) for each analysis of the depletion paradigm in Figure 3.

## Discussion

Across four studies, two with community samples and two with young adult samples, we found no evidence for the depletion effect, despite employing the depletion protocol and dependent measures of self-control that have shown consistently large effect sizes and have been the most frequently used in the literature. Interestingly, plotting the mean effect sizes for the four studies and two combined samples (see Figure 3), showed that the direction of the (non-significant) effects are actually the opposite of what would be expected from the literature for every analysis except one (Stroop young adults sample).

The strength model of self-control has generated a great deal of interest. In this model, self-control is viewed as a limited resource; this resource is depleted by efforts to inhibit a thought, emotion, or behavior. A number of studies, done primarily with college age students, have shown that exerting self-control in one situation results in poorer self-control on a variety of subsequent tasks [4]. Based on the conception of self-control as a limited resource, the field has begun to investigate ways in which the self-control resource may be enhanced. Practicing small acts of self-control are suggested to increase the self-control reserve, and hence a number of studies are now examining the long-term effects of practicing self-control on future success at changing behaviors, such as smoking [7] and weight loss [14]. Given the potential importance of this model for understanding and treating a variety of risky behaviors (e.g., smoking, unhealthy eating), it is critical that a reliable measure of the effects of depletion on self-control be identified.

In this series of four studies, using the most commonly used depletion paradigm and measures of self-control with the largest effect sizes, we found no evidence that completion of a depleting task led to decreases in self-control. Our first two studies were done with community adults. When no effect of depletion was seen in these studies, we considered the possibility that the age of the participants was affecting the outcome, since there is some evidence that the depletion effect may be specific to younger adults [5]. Thus we repeated these two studies using college age samples; again, we found no evidence that the depletion task affected subsequent self-control.

We considered several possible explanations for these null results. One possibility is that we did not use the appropriate tasks. However, we selected crossing out letters as the depletion task because it is the most frequently used task (used in 20 studies), with the most consistently large effect sizes: an averaged corrected standardized difference effect size of d = 0.77 [4]. In addition, we felt that with crossing out letters, both the depletion and the control task could be administered consistently across subjects, whereas other depleting tasks, such as affect regulation while watching a video might vary across subjects who differed in their reaction to the specific video. We also selected the two most frequently used dependent tasks—handgrip and modified Stroop and administered these using carefully controlled protocols, including, as noted above, an updated protocol for handgrip persistence.

Another possible explanation for our null results is that we had insufficient power to detect a depletion effect. However, the sample size used in our studies is comparable to that used in many prior studies [4] and the use of a repeated measures design increases power within this sample size. Post-hoc analyses suggest that we had sufficient power to reject the null hypothesis. Specifically, we conducted a sensitivity analysis to detect the required effect size for each paradigm at 80% power (testing the within-between interaction), given the sample size and correlation between measures demonstrated in the current study. For the handgrip test, given our combined sample size of 102 and a correlation between measures of.74, the required effect size was d = .22. For the Stroop test, given our combined sample of 83 and a correlation between measures of.80, the required effect size was d = .20. Thus, the current study was adequately powered to detect the previously-reported effect sizes for handgrip and Stroop (d = .63 and d = .076, respectively, from a recent meta-analysis) [4].

The approach we used, in which pre- and post-measures were obtained for subjects who were randomly assigned to a depletion or control condition, is a stronger design than used in many of the prior studies in this area. Multiple studies have utilized non-experimental designs to investigate trait measures of self-control on task performance [11]; [15]–[20]. In some cases, studies involve pre and post assessments completed only on subjects exposed to depletion (i.e. there was no control group that was not depleted) [21]. Without a control group, the decreases in handgrip persistence we observed over time might be taken, incorrectly, as evidence of a depletion effect. In other studies, assessments are conducted only after depletion or control, without a pre-depletion assessment of the dependent measure [22]–[26]. Again, this could lead to erroneous interpretation of results.

While other possible explanations for our null results can be advanced, we suggest that our data may indicate that depletion has more limited effects on self-control than implied by the publications in this field. That is, as Carter & McCullough [6], [27] have suggested, the depletion effect may be overestimated in the literature due in part to publication bias. Currently there are few papers in the literature that presents null depletion findings [28], [29]

We would like to encourage publication of other studies using depletion paradigms—regardless of whether the results were positive, negative, or null. We recognize and support the goal of replication of results in psychological experiments, and feel that this is an area in need of such endeavors.

## Supporting Information

Data S1 De-identified data handgrip studies.
(XLSX)

Data S2 De-identified data for Stroop studies.
(XLSX)
